# Atm reactivation reverses ataxia telangiectasia phenotypes in vivo

**DOI:** 10.1038/s41419-018-0357-8

**Published:** 2018-02-22

**Authors:** Sara Di Siena, Federica Campolo, Roberto Gimmelli, Chiara Di Pietro, Daniela Marazziti, Susanna Dolci, Andrea Lenzi, Andre Nussenzweig, Manuela Pellegrini

**Affiliations:** 1 Department of Anatomical, Histological, Forensic and Orthopaedic Sciences, Sapienza University, Rome, Italy; 2 Department of Biomedicine and Prevention, Tor Vergata University, Rome, Italy; 3 Institute of Cell Biology and Neurobiology, CNR, Monterotondo, Rome, Italy; 4 Department of Experimental Medicine, Sapienza University, Rome, Italy; 5 Laboratory of Genome Integrity, National Cancer Institute, NIH, Bethesda, MD 20893 USA; 6 Department of Medicine and Health Science ‘V. Tiberio’, University of Molise, Campobasso, Italy

## Abstract

Hereditary deficiencies in DNA damage signaling are invariably associated with cancer predisposition, immunodeficiency, radiation sensitivity, gonadal abnormalities, premature aging, and tissue degeneration. ATM kinase has been established as a central player in DNA double-strand break repair and its deficiency causes ataxia telangiectasia, a rare, multi-system disease with no cure. So ATM represents a highly attractive target for the development of novel types of gene therapy or transplantation strategies. Atm tamoxifen-inducible mouse models were generated to explore whether Atm reconstitution is able to restore Atm function in an Atm-deficient background. Body weight, immunodeficiency, spermatogenesis, and radioresistance were recovered in transgenic mice within 1 month from Atm induction. Notably, life span was doubled after Atm restoration, mice were protected from thymoma and no cerebellar defects were observed. Atm signaling was functional after DNA damage in vivo and in vitro. In summary, we propose a new Atm mouse model to investigate novel therapeutic strategies for ATM activation in ataxia telangiectasia disease.

## Introduction

Mutations in the ataxia telangiectasia mutated (*ATM*) gene cause ataxia telangiectasia (A-T) syndrome, a rare disease that exhibits cancer predisposition, neurodegeneration, immunodeficiency, and premature aging of the skin and hair^1–3^. Many patients also exhibit somatic growth retardation and growth factor deficiency^4,5^. The disease is progressive, and death, caused predominantly by lung failure and cancer, generally occurs by the second or third decade of life^6^. At present, only symptomatic therapies are available and no cure for A-T syndrome exists.

The majority of A-T patients have mutations in *ATM*gene that result in complete loss of the protein^7^. ATM protein belongs to phosphatidylinositol 3-kinases family, including A-T and Rad3 related (ATR) and DNA-dependent protein kinase (DNA-PK) that share homologies within the catalytic domain. ATM is a key player in cell cycle checkpoint control and DNA repair after DNA double-strand breaks (DSBs) caused by endogenous sources, ionizing radiation (IR) or oxidative damage^8^. In response to DNA damage, >700 protein targets recognized by ATM and ATR have been identified^9^. Its deficiency impairs the signaling cascade indispensable for maintaining the genomic stability. Potential therapeutic approaches for the disease have been proposed, including treatments with myo-inositol, the use of antioxidants, or growth hormones supplementation^10^. Recently, a randomized trial showed that a short-term oral administration of the glucocorticoid, betamethasone, reduced ataxia symptoms in young A-T patients^11^. One promising therapeutic approach is bone marrow transplantation (BMT)^12–14^. BMT is already used as curative treatment for some genomic instability syndromes^15,16^, however, no transplantation protocols exist for A-T patients and transplantation therapy has been poorly applied^17–19^. Another interesting preclinical approach is the mutation-targeted therapy that ameliorates the *ATM* gene function through the aminoglycoside-induced readthrough of premature termination codons and the use of antisense morpholino oligonucleotides^20^.

The possibility of reinserting ATM protein or correcting point mutations as soon as the disease is diagnosed can be predicted to be of great benefit for A-T patients; however, whether and to what extent A-T phenotypes can be rescued, once ATM functions are restored, need to be tested in preclinical experiments. Atm null mice recapitulate most of the characteristics of A-T patients including extreme radiosensitivity, immune system deficiency, germ cell defects, and cancer predisposition^21–23^. They also show several cerebellar defects despite they do not exhibit neurodegeneration^21,24,25^. In this context, we generated a tamoxifen-inducible Atm mouse model and we asked whether wild-type Atm restoration is able to rescue the immunological, neurological and reproductive defects, improve weight gain, induce radioresistance, prevent and/or reduce tumor progression and whether these events depend on the timing of Atm reactivation. The rescue of Atm null phenotypes is shown at different timing after Atm induction and we unveil the cellular and molecular mechanisms controlling these events.

## Results

### Generation of transgenic mice expressing the tamoxifen-inducible Atm kinase

**Fig. 1:**
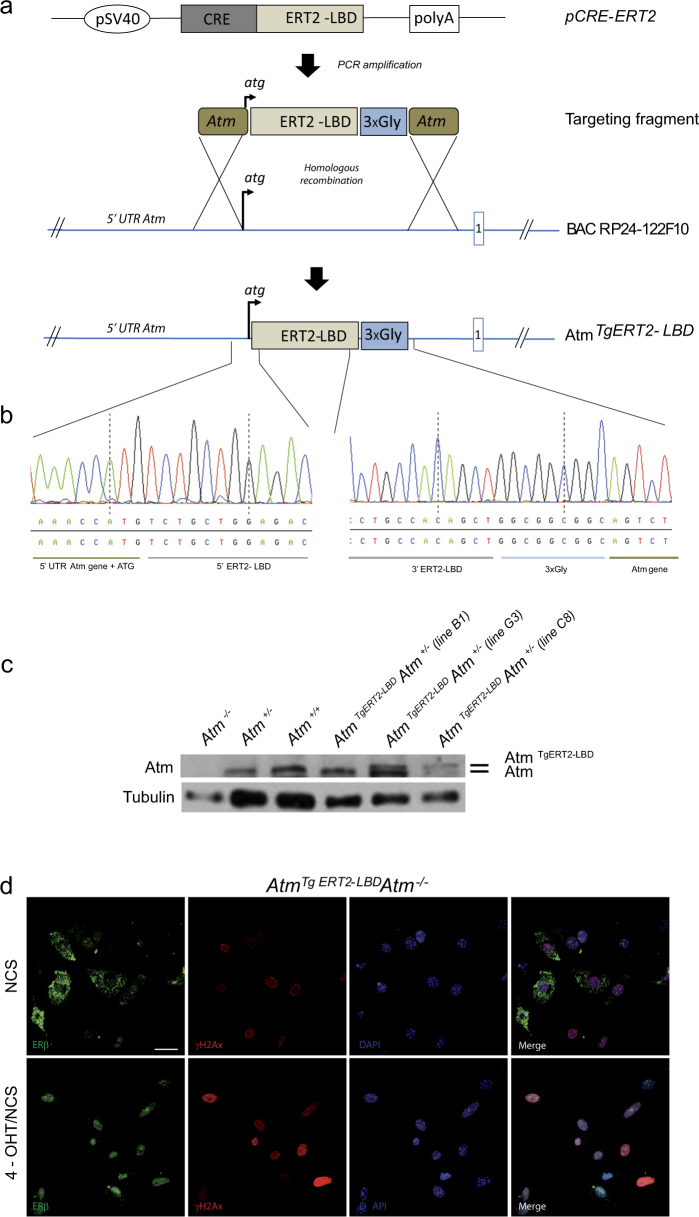
Generation of *Atm*^*TgERT2-LBD*^ transgenic mouse model. **a** Schematic representation of BAC construct for the generation of mouse models carrying a tamoxifen-inducible version of wild-type Atm. The *pCRE-ERT2* plasmid was used to amplify the modified mouse estrogen receptor ligand-binding domain (ERT2-LBD). Primers for ERT2-LBD amplification were flanked by 100 bp Atm oligos surrounding the ATG site. Three glycine residues were introduced between the 3′ end of LBD and the first amino acid of Atm to ensure the correct protein folding. The Atm-ERT2-LBD-Atm fragment was inserted into the wild-type Atm BAC RP24-122F10 by homologous recombination to obtain an *Atm*^*Tg ERT2-LBD*^ BAC. The first intron is reported in the scheme. **b** Sequence chromatogram showing the correct insertion of *Atm-ERT2-LBD-Atm* fragment in the murine *Atm* BAC. **c** Western blot analysis showing Atm^Tg ERT2-LBD^ expression in thymocytes of two founder lines (G3 and C8) crossed to *Atm*^*+/−*^ mice. Endogenous Atm (lower band) and transgenic Atm (upper band) are shown. **d** Immunofluorescence on *Atm*^*Tg ERT2-LBD*^ *Atm*^*−/−*^ embryonic mouse fibroblasts treated or not for 24 h with 4-OHT and damaged with NCS 500 ng/ml for 10 min. ERβ staining (green) to detect Atm and γ-H2AX (red) are shown. DAPI was used to stain nuclei. Scale bar 50 μm

To spatially and temporally control Atm reactivation in vivo, we generated a mouse model carrying an activatable version of wild-type Atm, by using a bacterial artificial chromosome (BAC) recombineering strategy (Fig. 1). A sequence encoding a modified ligand-binding domain of the mouse estrogen receptor, named ERT2-LBD, which binds tamoxifen, but not endogenous estrogens, was fused in-frame to the coding sequences of wild-type Atm. The fusion was carried out in the BAC RP24 122F10 vector, containing *Atm* gene^26–28^ (Fig. 1a). BAC modification was confirmed by sequencing analysis before the generation of transgenic mice (Fig. 1b). Founder lines expressing ERT2-LBD-Atm were identified and bred to *Atm*^*+/−*^ for two generations to obtain *Atm* ^*TgERT2-LBD*^ *Atm*^*−/−*^. Founder lines C8 and G3 were found to express ERT2-LBD-Atm protein near the level of 25% and 50% of *Atm*^*+/+*^ mice, respectively (Fig. 1c). G3 line was then analyzed in detail.

In the absence of 4-hydroxytamoxifen (4-OHT), a tamoxifen metabolite, ERT2-LBD-Atm fusion kinase remained sequestered into the cytoplasm (Fig. 1d) and no Atm-dependent response to the DNA damage inducer neocarzastatin (NCS) was observed in embryonic murine fibroblasts (MEFs), thymocytes and ear fibroblasts (EFs), isolated from *Atm* ^*TgERT2-LBD*^*Atm*^*−/−*^ mice (Fig. 1d and Fig. S1a, b). Administration of 1 μM 4-OHT for 24 h elicited Atm nuclear translocation and activation following DSBs (Fig. 1d and Fig. S1b). After 4-OHT treatment, Atm signaling was restored in *Atm* ^*TgERT2-LBD*^ *Atm*^*−/−*^ EFs damaged with bleomycin, a single- and double-strand DNA damage inducer and this response was partially counteracted by KU55933, a selective Atm inhibitor (Fig. S1c). More consistently, transgenic MEFs, treated overnight with 1 μM 4-OHT before γ-irradiation, activated an Atm-dependent signaling cascade similarly to *Atm*^*+/−*^ MEFs (Fig. S1d). Atm and DNA-PK inhibitors were used to confirm the selectivity of Atm-dependent signaling.

Altogether, these results show that we generated a transgenic mouse model in which Atm is inactive and acquire responsiveness to DNA damage after 4-OHT treatment in vitro.

### Atm reactivation improves body growth and T-cell development

**Fig. 2:**
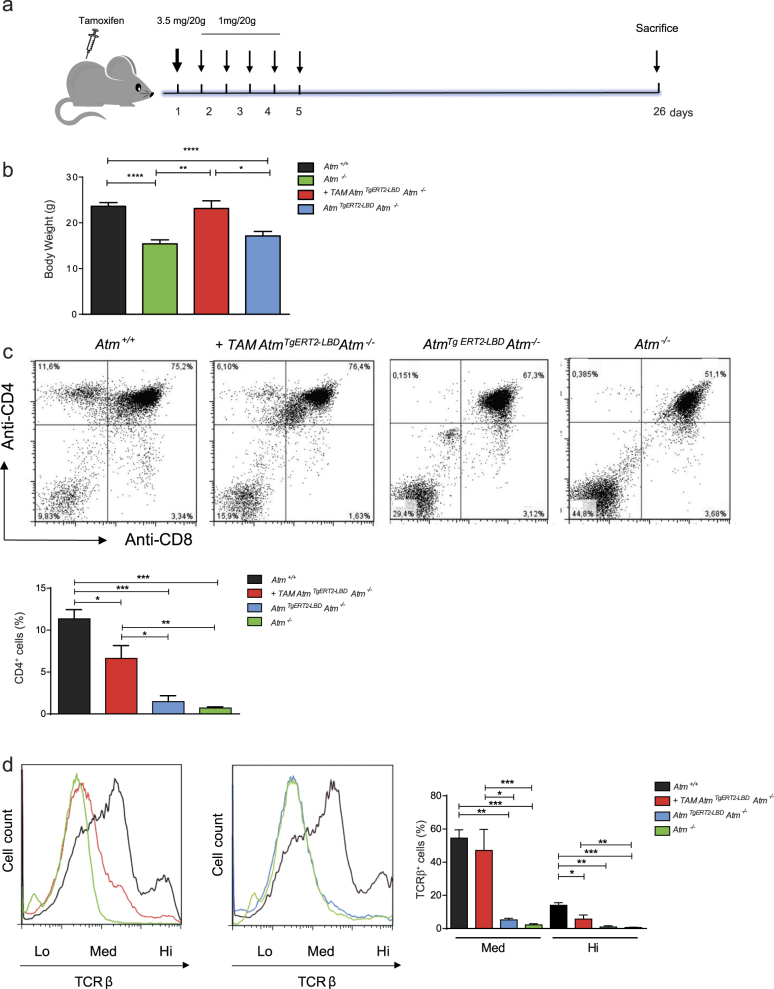
Activation of Atm^TgERT2-LBD^ partially reconstitutes lymphocyte development. **a** Flow chart of the strategy used to restore Atm expression in vivo. Forty-five days old mice were treated with tamoxifen by intraperitoneal injections as shown. Transgenic mice were sacrificed 26 days after the injection. Doses and timing of tamoxifen treatment were described in Materials and methods section. **b** Histogram showing body weight of *Atm*^*+/+*^, *+*TAM *Atm*^*Tg ERT2-LBD*^*Atm*^*−/−*^, *Atm*^*Tg ERT2-LBD*^*Atm*^*−/−*^, and *Atm*^*−/−*^ mice measured at the end of experimental protocol (*n* = 5 mice for each genotype). *P < 0.05, **P < 0.01, and ****P < 0.0001. **c** Representative flow cytometric analysis of surface expression of CD4 versus CD8 in freshly isolated thymocytes form *Atm*^*+/+*^, +TAM *Atm*^*Tg ERT2-LBD*^*Atm*^*−/−*^, *Atm*^*Tg ERT2-LBD*^*Atm*^*−/−*^, and *Atm*^*−/−*^ mice. Histogram shows the percentage of CD4 single-positive cells. Data are representative of at least *n* = 4 thymocyte preparations for each genotype in independent experiments. *P < 0.05, **P < 0.01, and ***P < 0.001. **d** Representative flow cytometry analyses of TCR-β surface marker expression in *Atm*^*+/+*^*, +*TAM *Atm*^*Tg ERT2-LBD*^*Atm*^*−/−*^, *Atm*^*Tg ERT2-LBD*^*Atm*^*−/−*^, and *Atm*^*−/−*^ mice. The dotted line shows TCR-β expression (low, medium, and high) in freshly isolated thymocytes. Histogram shows the percentage of medium and high TCR-β-expressing cells. Data were obtained from independent experiments performed on at least *n* = 4 thymocyte preparations for each genotype in independent experiments. *P < 0.05, **P < 0.01, and ***P < 0.001

Atm reactivation was achieved in vivo by intraperitoneal injection of tamoxifen to 45 days old *Atm* ^*TgERT2-LBD*^ *Atm*^*−/−*^ mice (Fig. 2). *Atm*^*−/−*^ and *Atm*^*+/+*^ mice were also treated with tamoxifen and no toxicity of the drug was observed.

Mice were sacrificed 26 days after the injections (Fig. 2a). Tamoxifen administration rescued the body size of *Atm* ^*TgERT2-LBD*^ *Atm*^*−/−*^ mice compared with *Atm*^*−/−*^ and untreated transgenic mice (Fig. 2b).

We then investigated thymocyte maturation in *Atm* ^*TgERT2-LBD*^ *Atm*^*−/−*^ mice treated or not with tamoxifen (Figs. 2c, d). In the developing thymus, Atm functions in T-cell receptor (TCR)-α gene rearrangement by facilitating the resolution of DSBs, which in turn promotes TCR-β surface marker expression and CD4 and CD8 single-positive (SP) thymocyte selection^29,30^. The physiological role of Atm reactivation was at first examined by flow cytometry on freshly isolated thymocytes from *Atm* ^*+/+*^, *Atm*^*−/−*^ and *Atm* ^*TgERT2-LBD*^ *Atm*^*−/−*^ mice (Figs. 2c, d).

Analysis of T-cell development revealed that the percentage of CD4-positive cells was partially recovered after tamoxifen in transgenic mice compared with knockout mice, but not in transgenic mice that were not treated with tamoxifen (Fig. 2c). Moreover, the percentage of the high and medium TCR-β-expressing T cells was increased after tamoxifen administration compared with *Atm*^*−/−*^ and untreated transgenic mice (Fig. 2d).

Altogether, these results indicate that Atm reactivation is able to recover body growth and T-cell development.

### Atm reactivation restores male germ cell maturation and fertility

*Atm*^*−/−*^ males and females are infertile because of chromosomal fragmentation and impairment of meiosis^21–23^, the cellular process that controls gamete differentiation. The meiotic process starts during embryogenesis in females and establishes the oocyte pool, whereas it occurs postnatally in males and it is a continuous process that lasts lifelong.

**Fig. 3:**
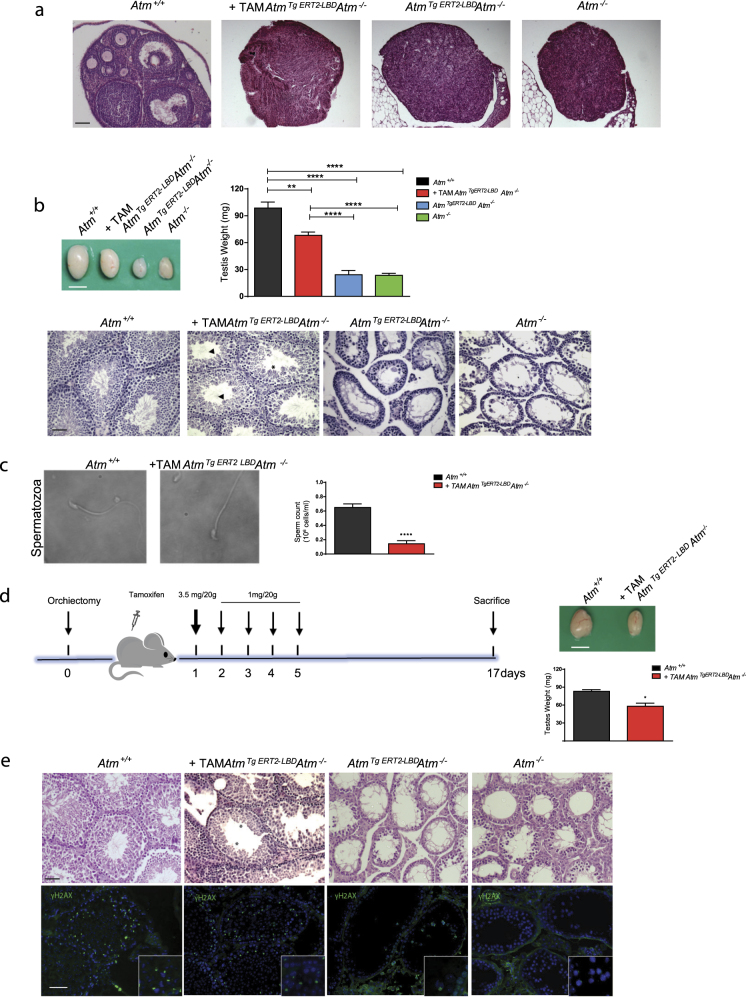
Atm reactivation in *Atm*^*TgERT2-LBD*^*Atm*^*−/−*^ mice reconstitutes male germ cell development. **a** Representative image of ovaries sections stained with H&E *n* = 3 females analyzed for each genotype. Scale bar = 100 μm. **b** Testis picture of two months *Atm*^*+/+*^*, +*TAM *Atm*^*Tg ERT2-LBD*^*Atm*^*−/−*^, *Atm*^*Tg ERT2-LBD*^*Atm*^*−/−*^, and *Atm*^*−/−*^ old mice. Scale bar = 5 mm. Histogram shows the mean value ± SEM of testis weight (*n* = 5 mice for each group). **P < 0.01 and ****P < 0.0001. H&E staining highlighted the presence of elongated spermatids (asterisks) and also spermatozoa (black arrowheads). Scale bar = 50 μm. **c** Epididymal spermatozoa pictures isolated from two months old *Atm*^*+/+*^ and +TAM *Atm*^*Tg ERT2-LBD*^*Atm*^*−/−*^mice. Images were captured in white field at the Nikon Eclipse Ti-S microscope. Scale bar = 10 μm. Histogram of sperm count is shown as mean value ± SEM (*n* = 6 mice for each group). ****P < 0.0001. **d** Flow chart of Atm restoration in 45 days old mice treated with tamoxifen and sacrificed 17 days after the injection. Testes picture and testis weight at day 17 is shown. Scale bar = 5 mm. *P < 0.05. **e** H&E section staining of *Atm*^*+/+*^, *+*TAM *Atm*^*Tg ERT2-LBD*^*Atm*^*−/−*^,+ORCH *Atm*^*Tg ERT2-LBD*^ *Atm*^*−/−*^, and *Atm*^*−/−*^ testes. Asterisks show round and elongated spermatids in +TAM *Atm*^*Tg ERT2-LBD*^*Atm*^*−/−*^ seminiferous tubules. Immunofluorescence for H2AX phosphorylation was performed in adjacent sections. Scale bar = 50 μm. Magnification shows γ-H2AX staining in sex body of pachytene spermatocytes. DAPI was used to stain nuclei

*Atm* ^*TgERT2-LBD*^ *Atm*^*−/−*^ female were sterile and showed small ovaries without mature oocytes also after tamoxifen treatment resembling *Atm*^*−/−*^ ovaries (Fig. 3a). This result confirm that the Atm transgene cannot be activated by endogenous estrogens and more widely that female meiosis cannot be rescued postnatally. Conversely, *Atm* ^*TgERT2-LBD*^ *Atm*^*−/−*^ males showed a consistent testis size increase compared with *Atm*^*−/−*^ males due to the presence of mature germ cells after Atm induction with tamoxifen (Fig. 3b). As expected, germ cell degeneration and complete absence of spermatids was found in testes from untreated *Atm* ^*TgERT2-LBD*^ *Atm*^*−/−*^ mice, excluding the possibility that transgenic males variably produced mature germ cells (Fig. 3b).

Sperm collection from caudae 26 days after tamoxifen injection revealed the presence of spermatozoa with normal shape and motility (Fig. 3c), although the cell number was reduced (*n* = 0.143 ± 0.043 × 10^6^ / ml in reactivated *Atm* ^*TgERT2-LBD*^ *Atm*^*−/−*^ vs 0.65 ± 0.05 × 10^6^ / ml in *Atm* ^+/+^).

To test whether inducible Atm was acting on meiotic spermatocytes arrested in prophase, tamoxifen-induced *Atm* ^*TgERT2-LBD*^ *Atm*^*−/−*^ males, which either underwent or not mono-orchiectomy, were sacrificed 17 days after injections (Fig. 3d). This is the shortest time needed for spermatids differentiation from meiotic cells. The analysis of testis after orchiectomy (ORCH) showed a knockout phenotype (data not shown), whereas testis size was increased (Figs. 3b, d) and round and elongated spermatids were found (Fig. 3e, upper panels), 17 days after tamoxifen injections in seminiferous tubules of the contralateral testis, indicating that reactivated Atm was likely acting on meiotic prophase cells. Atm activation was confirmed by H2AX phosphorylation (γ-H2AX), a target of Atm in the sex body region of pachytene spermatocytes (Fig. 3e, lower panels). Untreated transgenic mice showed few γ-H2AX-positive cells similarly to *Atm*^*−/−*^ mice. Notably, transgenic males were able to fertilize females and to give pups within 2 months of continuous breeding after tamoxifen treatment (Fig. S2). The fertility rescue was restricted to this window most likely due to completion of the spermatogenetic wave. Collectively, our results show that physiological DNA rearrangements that resolve DSBs during meiotic recombination are rescued in the Atm-inducible male mouse model and they likely occur during the early stages of meiotic germ cell differentiation.

### Atm restoration induces physiological responses to IR and Atm signaling cascade

**Fig. 4:**
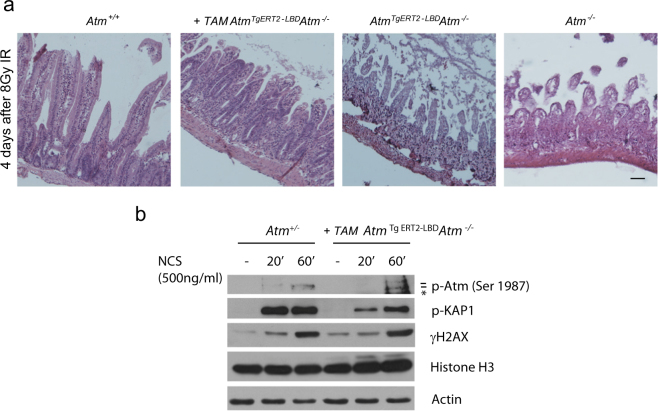
DNA damage signaling reactivation in tamoxifen-treated *Atm*^*TgERT2-LBD*^*Atm*^−/−^ mice. **a** Representative image of small intestine sections stained with H&E. Mice were irradiated with 8 Gy and sacrificed 4 days after treatment. Scale bar = 100 μm. **b** Western blot of thymocytes collected from *Atm*^*+/−*^ and *Atm*^*TgERT2-LBD*^*Atm*^−/−^ mice 26 days after tamoxifen treatment. Cells were freshly isolated and exposed to NCS. Atm, Kap1, and H2AX phosphorylation are shown. Asterisk indicates a not specific band. Data are representative of two independent experiments

*Atm*^*−/−*^ mice have an increased sensitivity to IR^31^. Upon radiation exposure, the death of *Atm*^*−/−*^ mice results from acute radiation toxicity to the gastrointestinal tract^21^. *Atm* ^*TgERT2-LBD*^ *Atm*^*−/−*^ mice with or without tamoxifen administration were subjected to 8 Gy of whole-body γ-irradiation to evaluate the radiation sensitivity. Intestinal tissues were collected 4 days after irradiation exposure. Recovery from irradiation in the small intestines was similar in *Atm*^*+/−*^ and *Atm* ^*TgERT2-LBD*^ *Atm*^*−/−*^ after tamoxifen treatment, whereas *Atm*^*−/−*^ and *Atm* ^*TgERT2-LBD*^ *Atm*^*−/−*^ mice displayed characteristic toxicity outlined by severe epithelial crypt degeneration and loss of villi (Fig. 4a). These data indicate that Atm-inducible mouse model is able to promote DNA repair and tissue recovery after exposure to IR.

To study if cells were still able to respond in vitro to DNA damage after Atm recovery in vivo, thymocytes isolated from tamoxifen-treated *Atm* ^*TgERT2-LBD*^*Atm*^*−/−*^ mice were damaged with NCS. As shown in Fig. 4b, Atm signaling cascade was activated similarly to *Atm*^*+/−*^ thymocytes in response to DNA damage.

These results confirm that tamoxifen treatment drives Atm unfolding allowing Atm nuclear re-localization in an activatable state prone to respond to DNA damage.

### Atm reactivation prevents/delays thymic lymphoma in *Atm*^*−/−*^ mice

**Fig. 5:**
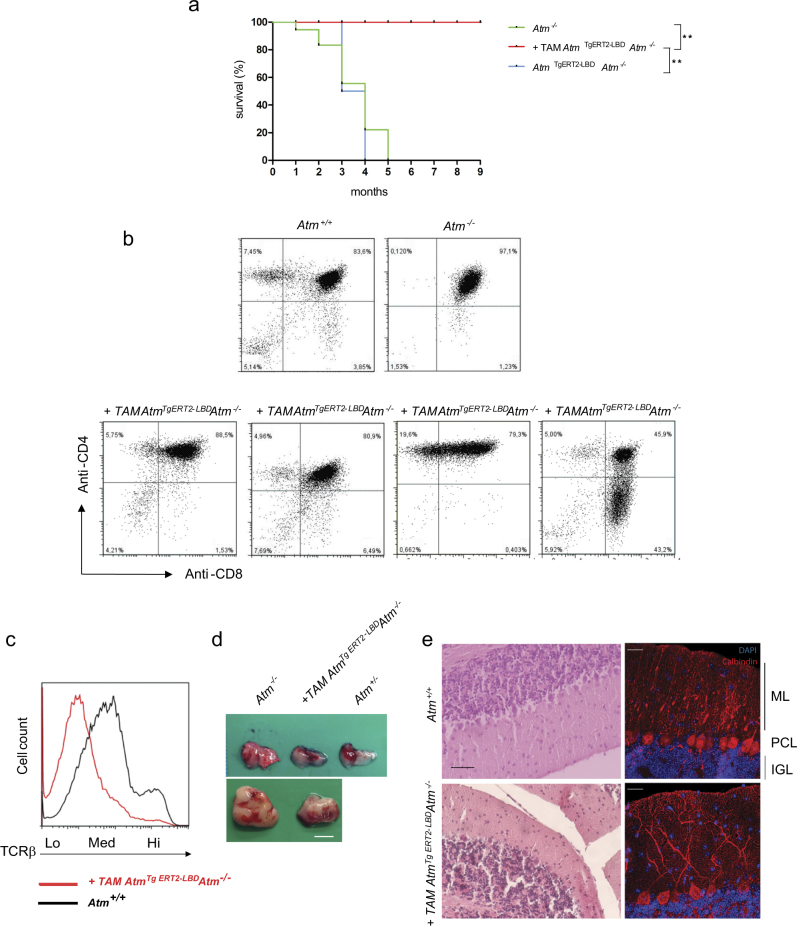
In vivo long-term responses of *Atm*^*TgERT2-LBD*^*Atm*^−/−^ mice to tamoxifen. **a** Kaplan–Meier curves of 45 days *Atm*^TgERT2-LBD^*Atm*^−/−^ old mice that were either treated with vehicle (*n* = 4), or tamoxifen (*n* = 6) compared with *Atm*^−/−^ (*n* = 6) mice. *P*-value was calculated with the Mantel–Cox log-rank test. **P < 0.01. **b** Flow cytometry analysis on thymocytes collected from thymus of 9 month old mice. Representative CD4 and CD8 expression pattern in *Atm*^*TgERT2-LBD*^*Atm*^−/−^ mice after tamoxifen treatment is shown. Data were obtained from four independent experiments performed on at least *n* = 6 mice of each genotype. **c** Representative picture of normal (upper panel) and pathological (lower panel) *Atm*^*TgERT2-LBD*^*/Atm*^−/−^ thymi collected at 9 months after tamoxifen treatment. Scale bar = 5 mm. Early (2 months) and late (4 months) thymoma of *Atm*^−/−^ mice are shown for comparison. **d** Representative flow cytometry analyses of TCR-β expression. The dotted line shows TCR-β expression (low, medium, and high). Data were obtained from independent experiments performed on two mice. **e** Representative H&E staining of cerebellar sections of *Atm*^*TgERT2-LBD*^*Atm*^−/−^ mice treated with tamoxifen and their *Atm*^+/+^ littermate (left panels). Scale bar = 100 μm. Immunofluorescence labeling of calbindin (red) to identify Purkinje cells in *Atm*^*TgERT2-LBD*^*Atm*^−/−^ and *Atm*^+/+^ sections (right panels). Molecular (ML), Purkinje Cell (PGL), and Internal Granule (IGL) layers are shown. Nuclei were stained with DAPI (blue). Scale bar = 25 μm. Experiments were performed on *n* = 3 mice of each genotype

To understand whether Atm reactivation rescues Atm-null phenotypes in the long-term, *Atm* ^*TgERT2-LBD*^ *Atm*^*−/−*^ mice were monitored for several months after tamoxifen injections. *Atm*^*−/−*^ mutant mice acquire fatal thymic malignancies as early as 2 months of age and, by 5 months of age, *Atm*^*−/−*^ mutant mice die for thymic lymphomas with some variability depending on the housing conditions^21^. In our animal facility, *Atm*^*−/−*^ mice die within 5 months of age (Fig. 5a). Similarly, to *Atm*^*−/−*^ mice, *Atm* ^*TgERT2-LBD*^*Atm*^*−/−*^ mice without tamoxifen injection succumbed for thymic lymophomas at similar age (Fig. 5a). Notably, Atm-inducible transgenic mice survived at least 9 months, a time at which we decided to sacrifice them for further analyses.

Atm-induced *Atm* ^*TgERT2-LBD*^*Atm*^*−/−*^ mice were analyzed at 9 months of age for development of mature T cells, evaluating CD4 and CD8 markers, TCR-β expression and thymoma formation (Figs. 5b–d and Fig. S3). As reported in Figs. 5b, c, around 50% of the mice showed T-cell maturation over the time and did not develop thymoma by this age. The other 50% presented tumoral expression pattern of CD4 and CD8 markers and showed thymoma (Figs. 5b, d and Fig. S3a), although reduced in size. Peripheral blood analyses revealed that double-positive CD3/CD4 cells were increased in induced *Atm* ^*TgERT2-LBD*^*Atm*^*−/−*^ mice compared with *Atm*^*−/−*^ (Fig. S3b), suggesting that mature T-cell pool is maintained for a long period after tamoxifen treatment.

### Atm restoration rescues Purkinje cell defects

*Atm*^*−/−*^ mice do not show signs of neurodegeneration during their life span^32^, however, several studies reported that cerebellum of A-T mouse models surviving longer than 6 months had reduced cerebellum size index^13^, alteration of synaptic vesicle formation and release^33^, ectopic and abnormally differentiated Purkinje cells^13,25^. These defects are frequently observed in A-T patients^34^. Inducible Atm transgenic mice similarly to *Atm*^*−/−*^ mice did not show evident cerebellar phenotypes within 5 months of age (data not shown). To investigate whether reactivation of Atm was sufficient to prevent cerebellum abnormalities in older transgenic mice, we analyzed 9 months old *Atm* ^*TgERT2-LBD*^*Atm*^*−/−*^ mice that were treated with tamoxifen at day 45. Histological examination (Fig. 5e, left panels) and immunofluorescence analyses (Fig. 5e, right panels) did not reveal major differences in the cerebella and in Purkinje cell morphology and number between *Atm*^*+/+*^ and *Atm* ^*TgERT2-LBD*^*Atm*^*−/−*^ mice suggesting that transgene expression preserved cerebella integrity in an *Atm* knockout background.

During the timing of survival test, male mice were bred with females of different backgrounds, but no pups were born 2 months after tamoxifen treatment. Indeed, mice analyzed at 9 months showed small testes and seminiferous tubules were depleted of meiotic and post meiotic germ cells (Fig. S4).

Overall, these results indicate that one single series of injections performed in 45 days old mice is able to prevent neuronal defects, sufficient to induce sperm cell production, recover male fertility in a short time span, and more importantly it is capable to prolong *Atm*^*−/−*^ mouse survival by restoring thymocyte development and preventing/delaying thymoma formation.

## Discussion

A-T is a highly pleiotropic autosomal recessive human disorder that is caused by mutations in the *ATM* gene, located on the long arm of chromosome 11 (q22–23)^1,2^. A-T patients exhibit thymic hypoplasia, resulting in immunodeficiency and hematologic malignancy, neurodegeneration, sterility, and radiosensitivity^3^. Here, we report that transgenic mice carrying a regulatable *Atm* gene fused to the modified estrogen receptor ligand-binding domain (ERT2-LBD), rapidly and reversibly activate the kinase following administration of tamoxifen, the ERT2 ligand. Several proteins have been successfully regulated by ERT2/tamoxifen system (see http://www.picard.ch/downloads for protein list), and useful conditional mouse models have been recently generated^35,36^. However, this is the first mouse model in which ERT2 has been fused to Atm protein, allowing to directly and temporally modulate its activation. Notably, a single series of injections, expected to activate Atm just in a limited period of time, was sufficient to induce thymocyte and sperm maturation, to increase body weight, to rescue germ cell, and cerebellum differentiation and more widely to maintain genomic stability and prevent/delay tumorigenesis.

Meiotic germ cell arrest in *Atm*^*−/−*^ mice has been extensively studied^37^. Interestingly, we found that Atm reactivation in the meiotic phase was sufficient to restore male fertility, providing novel biological insights with potential implications in the reproductive medicine. *Atm* transgene might also regulate pre-meiotic germ cell survival, since pups were born within 2 months from the first tamoxifen injection, during continuous breeding. The limited temporal rescue of spermatogenesis and fertility of *Atm* ^*TgERT2-LBD*^ *Atm*^*−/−*^ mice can be explained by the relatively short half-life of tamoxifen in vivo^38,39^.

Interestingly, we observed a long-term restoration of thymocyte development. For example, the percentage of CD4 and CD8 SP cells reached wild-type levels 26 days after tamoxifen injection and these levels were maintained for several months. A possible scenario is that mature thymocytes produced following Atm reactivation exit from thymus, proliferate and then they return to the organ establishing a positive feedback loop. Studies on T-cell turnover indicate that most peripheral T cells can remain in interphase for months in rodents and years in humans^40,41^. Indeed mature CD4 and CD8 cells in the extrathymic environment are kept alive by TCR contact with self-peptide/major histocompatability complex (pMHC) ligands and exposure to interleukin-7^42,43^ before reentering into thymus where they are stored for long-term period^44^.

In previous studies of wild-type Atm BMT mouse models, the stem cells pool was described to account for thymocyte maturation and thymoma prevention^12–14^. Our data indicate that Atm restoration in inducible mice not older than 2 months was able to prevent/delay thymoma formation. We hypothesize that the long-term phenotypic rescue and tumor retardation in Atm reactivated mice is due to protection from TCR-associated translocations, which promotes thymomas. Furthermore, our data support previous hypothesis^12^ that a cell-intrinsic defect exists in T-cell progenitors as opposed to compromised microenvironment responsible for the inefficient T-cell maturation. Finally, we elucidated that malignancy protection is possible also in the case of Atm expression chimerism because in our model Atm-restored cells coexist with Atm-null cells once tamoxifen is degraded.

Altogether these results suggest that ATM restoration is theoretically a viable option for A-T patients; however, further studies are necessary to dissect the timing for tumor onset prevention or regression after tamoxifen treatment.

Exposure of mice to γ-irradiation revealed that reactivated Atm was able to repair DNA damage from exogenous sources other than physiological DSBs occurring during crossing over or TCR rearrangements. Small intestines appear reconstituted suggesting stem cell from crypt zone were activated upon tissue damage. Since mice were irradiated 3 weeks after tamoxifen treatment tissue repair was potentially driven by DNA-label-retaining cells, long-lived quiescent cells that represent a reservoir of stem cells in the small intestine^45^.

We further investigated whether the rescue of *Atm* knockout phenotypes was due to Atm signaling reactivation in response to DNA damage. Indeed, thymocytes collected from tamoxifen-induced transgenic mice were able to signal DNA damage in vitro.

Neurodegeneration is a detrimental hallmark of A-T disease caused by microscopic atrophy of the cerebellar cortex resulting from a reduction in Purkinje, granular, and basket cells. Atm-deficient mice do not show evident cerebellar defects presumably because they die at 4–6 months before their manifestation^21^. Indeed, recently, Atm mouse models in which survival was extended to 8–10 months showed that Atm-deficient mice have cerebellar defects^13,25^. To explain the later cerebellar phenotype it has been proposed that defective Atm might permit specific neuronal cell population, such as Purkinje cells to accumulate mutations that lead to functional deficits later in life^46^. Moreover, neurons of humans and mice at high risk for degeneration show clear evidence of having re-initiated a cell cycle process^47^, which is uncoupled from cell death^48^. Neurons do not complete the cell cycle and cell death is protracted for a long period that in mice last >6–12 months^49,50^. Our results show that Atm restoration in the second month of mouse life extended the survival at least for 9 months and mice did not present evident cerebellar defects. Since cell cycle events in mouse cerebellum occur primarily during the third postnatal week^49^, we hypothesize that by activating Atm before the second month of mouse life we prevented the ectopic Purkinje cell division causing neuronal abnormalities and thus cerebellum defects. Future work will focus on studying the time-dependent function of Atm on nervous system, by delaying Atm reactivation.

In summary, these results indicate that we were able to generate an Atm mouse model with the unique feature, compared with the other existent Atm mouse model, which Atm can be reactivated at any time during the mouse life span, permitting the temporal dissection of Atm functions in different cellular processes. Furthermore, this model is useful for understanding how long Atm functions can last in different cell types after the physiological turnover of the protein. In this context, our data showing that survival of Atm induced mice are consistently prolonged provide support for therapeutic interventions restoring ATM function in ATM-deficient cells and will permit future studies into the role of ATM in oxidative stress, cancer, and aging.

## Materials and methods

### Generation of *Atm*^TgERT2- LBD^ mice

The pCRE-ERT2^51,52^ was used to amplify the modified mouse ERT2-LBD, which responds to 4-OHT. The ERT2-LBD complementary DNA was amplified with expand long template taq (Roche, Monza, Italy), from the pCre-ERT2 plasmid^52^, using two 100-bp oligos surrounding Atm ATG site. Three glycine residues were introduced between the 3′ end of ERT2-LBD and the first amino acid of Atm to ensure the correct protein folding. The ERT2-LBD fragment was inserted into Atm BAC RP24 122F10^26^ by homologous recombination to obtain Atm^TgERT2-LBD^. The final targeting vectors were purified and diluted (10 μg/μl) for pronuclear injection into C57Bl/6 oocytes. The presence of the transgene was determined by screening tail DNA using BAC-specific PCR primer pairs, which amplify products in LBD sequence, 5′ end of Atm (LBD Screen For 5′-CAGCATGAAGTGCAAGAACG-3′ and AT Screen Rev 5′-AAAACAGCATCCCAATTCAG-3′). Several transgenic founder lines were obtained and crossed to *Atm* ^*+/−*^ mice in order to restore Atm expression in the Atm-null background. In the murine, *Atm* BAC was previously inserted an *Eco*RI site between 35 and 36 exons for a PCR-based method^28^ to distinguish between *Atm* ^*TgERT2-LBD*^ *Atm*^*+/−*^ and *Atm* ^*TgERT2-LBD*^ *Atm*^*−/−*^ genotypes.

Health status of the treated mice was monitored twice a week. Mice were housed at the animal facility of Tor Vergata University, under standard conditions with free access to food diet and water. All animal studies were performed in accordance with the Guidelines for the Care and Use of Laboratory Animals and protocols were approved by the Tor Vergata Animal Care, Ethics Committee and the Italian Ministry of Health (prot n 1104/2016-PR, art. 31 of D.lgs 24/2014).

### Tamoxifen and 4-OHT treatments

To induce Atm reactivation in vivo, 45 days old mice were injected with tamoxifen (Sigma, Milan, Italy) dissolved in corn oil (Sigma). Doses were chosen according to previous articles^36,53^ with some adjustments: *Atm* ^*TgERT2-LBD*^ *Atm*^*−/−*^ mice were treated with tamoxifen for 5 days (first day: 3.5 mg/20 g of body weight; following 4 days: 1 mg/20 g of body weight) by intraperitoneal injection.

Transgenic mice were sacrificed 17 or 26 days or 9 months after the tamoxifen injections.

*Atm*^*−/−*^ and *Atm*^*+/+*^ mice were also treated with tamoxifen as control mice with no evident effects of the drug. To induce Atm in vitro, cells were treated with 4-OHT (Sigma) dissolved in ethanol at different doses (from 10 nM to 2 μM) for 24 h.

### Cell isolation and western blot analysis

MEFs were derived from E13.5 *Atm* ^*TgERT2-LBD*^ *Atm*^*+/−*^ and *Atm* ^*TgERT2-LBD*^ *Atm*^*−/−*^ embryos by mincing embryonic carcass with sterile scalpels followed by digestion with 0.05% trypsin for 5 min, whereas to obtain mouse EFs the dorsal portion of the ear was minced and digested at 37 °C for 45 min in collagenase D/dispase (4 mg/ml in Dulbecco’s modified Eagle’s medium (DMEM), Roche). Cell suspensions were dissolved in DMEM containing 10% fetal bovine serum filtered and cultured for 48 h before use. MEFs and EFs were treated with 4-OHT (Sigma) for 24 h before stimulation with different DNA damage agents. DNA-PK inhibitor (NU7026:10 μM, Sigma) and ATM inhibitor (Kudo55933:10 μM, SelleckChem, Huston, TX, USA) were added to the medium 1 h before bleomycin (5 nM), NCS (500ng/ml, Sigma), and γ-irradiation (10 Gy) and cells were collected 1 h later, if not indicated.

T cells were isolated by gently smashing thymi in phosphate-buffered saline and then used for flow cytometry analyses or stimulated with NCS for 20 min or 1 h for protein analyses. Protein extraction was performed with lysis buffer (50 mM Tris-HCl pH7.5, 200 mM NaCl, 0.2% NP40, 50 mM β-glicerophosphate, 1% Tween 20, 0.5 M phenylmethylsulfonyl fluoride) and protease inhibitors (Sigma).

Western blots for Atm and protein targets were performed as previously described^26^. Primary antibodies were used overnight at the following dilutions: anti-mouse Atm S1987p (1:500), produced by immunization with the synthetic peptide SPTFEEGSpQGTTISS (Becton Dickinson)^26^, anti-Smc1 S957p (1:500, Rockland Immunochemicals, Limerick, PA, USA), anti-p53 S15p (1:500, Cell Signaling, Danvers, MA, USA), anti-p53 (1:1000, Santa Cruz, Dallas, TX, USA), anti-γH2AX (1: 1000 Upstate Biotechnology), and anti-Kap-1 S824p (1:700, Bethyl Labs, Montgomery, TX, USA), anti-tubulin (1:10,000, Sigma), anti-Actin (1:2000, Sigma), anti-Histone H3 (1:1000, Abcam, Cambridge, UK). Anti-rabbit or anti-mouse (1:10,000, Santa Cruz) were used as secondary antibodies. Bands were detected by ECL and images were recorded with the Syngene G-box system (Syngene Bioimaging, Haryana, India).

### ORCH, histological procedures and Immunofluorescence

ORCH of one testis was performed under general anesthesia with avertin (250 mg/kg, Sigma) 1 week before tamoxifen treatment. The abdominal cavity was opened to remove testis epydimis and part of the spermatic cord through the incision. The incision was closed with sutures. Mice received 500 µl of glucose solution (5% glucose in physiologic solution), soon after the surgery. Testes, ovaries, and small intestines were directly embedded in optical cutting temperature compound (BioOptica, Milan, Italy) and snap-frozen or previously fixed in Bouin solution (Sigma, Missouri, USA) and then included in paraffin. Sections 7 μm thick were stained with hematoxylin and eosin (H&E; Sigma, Missouri, USA) or processed for immunofluorescence with standard protocols. Images were acquired with an inverted microscope (Axiovert 200 M; Carl Zeiss, Inc., Thornwood, NY, USA). EFs cells or 5 μm thick testis sections were fixed in paraformaldehyde 4% (Società Italiana chimici, Rome, Italy) for 10 min at room temperature before immunofluorescence staining, using standard protocols. After permeabilization and blocking with 1% bovine serum albumin (BSA), samples were incubated with mouse anti-γH2AX (1:100 Upstate Biotechnology) and rabbit anti-ERβ (1:100, clone H-150, Santa Cruz) diluted in 0.5% BSA overnight at 4 °C. Secondary antibodies anti-mouse Alexa Fluor 488 or anti-mouse Alexa Fluor 568 or anti-rabbit Alexa Fluor 488 were diluted 1:400. Images were acquired by Nikon Eclipse Ti-S microscope (Nikon Instruments, Nikon Instruments S.p.A, Firenze, Italy).

Brain from 9-month-old mice were dissected and immediately fixed in paraformaldehyde 4% before paraffin embedding. Sections were processed for H&E staining with standard protocols or immunofluorescence labeling according to standard protocols with antigen retrieval. Calbindin 1 (Calb1/CalbD-28K; 1:200, clone CB-955; Sigma) was used as primary antibody and Alexa Fluor 555 as secondary antibody for Purkinje cell staining. Fluorescence micrographs were acquired with a TCS sp5 Leica laser scanning confocal microscope (Leica Microsystems, Milan, Italy), using the manufacturer’s imaging software. Experiments were performed in at least three animals for genotype. Sections of similar size in similar regions were chosen and analyzed in at least three non-adjacent sections per genotype.

### Flow cytometry

Single-cell suspensions of thymocytes were stained for 20 min with anti-CD4-PE, anti-CD8-FITC, or anti-TCR-β-APC antibodies (1:50; Miltenyi Biotec, Bologna, Italy) at 4 °C. Dead cells were stained with SYTOX® Blue (Thermo Fisher Scientific-Life Technologies, Waltham, MA, USA). Analysis was performed with CyAn cytofluorimeter (Dako, Milan, Italy) and analyzed using FlowJo software (version 7.2.4). Blood was collected from mouse eyes 1 week before the sacrifice and cells stained with anti-CD45-eFluor450 antibody (1:100; eBioscence, San Diego, CA, USA) to exclude red cells and with anti-CD3-APC, anti-CD4-PE, and anti-CD8-FITC antibodies (1:50; Miltenyi Biotec) to detect mature T cells.

### γ-Irradiation

For radiosensitivity experiments, mice were exposed to whole-body irradiation (8 Gy) using Gilardoni CHF 320 G X-ray generator (Gilardoni SpA, Lecco, Italy) operated at 250 kVp, 15 mA, with HVL = 1.6 mm Cu (additional filtration of 2.0 mm Al and 0.5 mm Cu). Mice were euthanized 4 days after irradiation.

### Statistical analyses

All data are expressed as mean ± SEM. Samples were analyzed with one-way analysis of variance with Tukey correction or with Student’s *t-*test, two tailed, and 1 degree of freedom. Differences were considered significant if *P < 0.05. Kaplan–Meier survival curves was plotted by using Graph Pad Prism version 6.0 (Graph pad software). *P*-values were obtained by the log-rank (Mantel–Cox) test.

## Electronic supplementary material

Supplemental figures
