# Supplementary material for: Atm reactivation reverses ataxia telangiectasia phenotypes in vivo
Source: Cell Death Dis. 2018 Feb 22;9(3):314. doi: 10.1038/s41419-018-0357-8 (PMC5833483; doi:10.1038/s41419-018-0357-8)
Supplement: Supplementary file 1 — Supplemental figures [file 41419_2018_357_MOESM1_ESM.pdf]

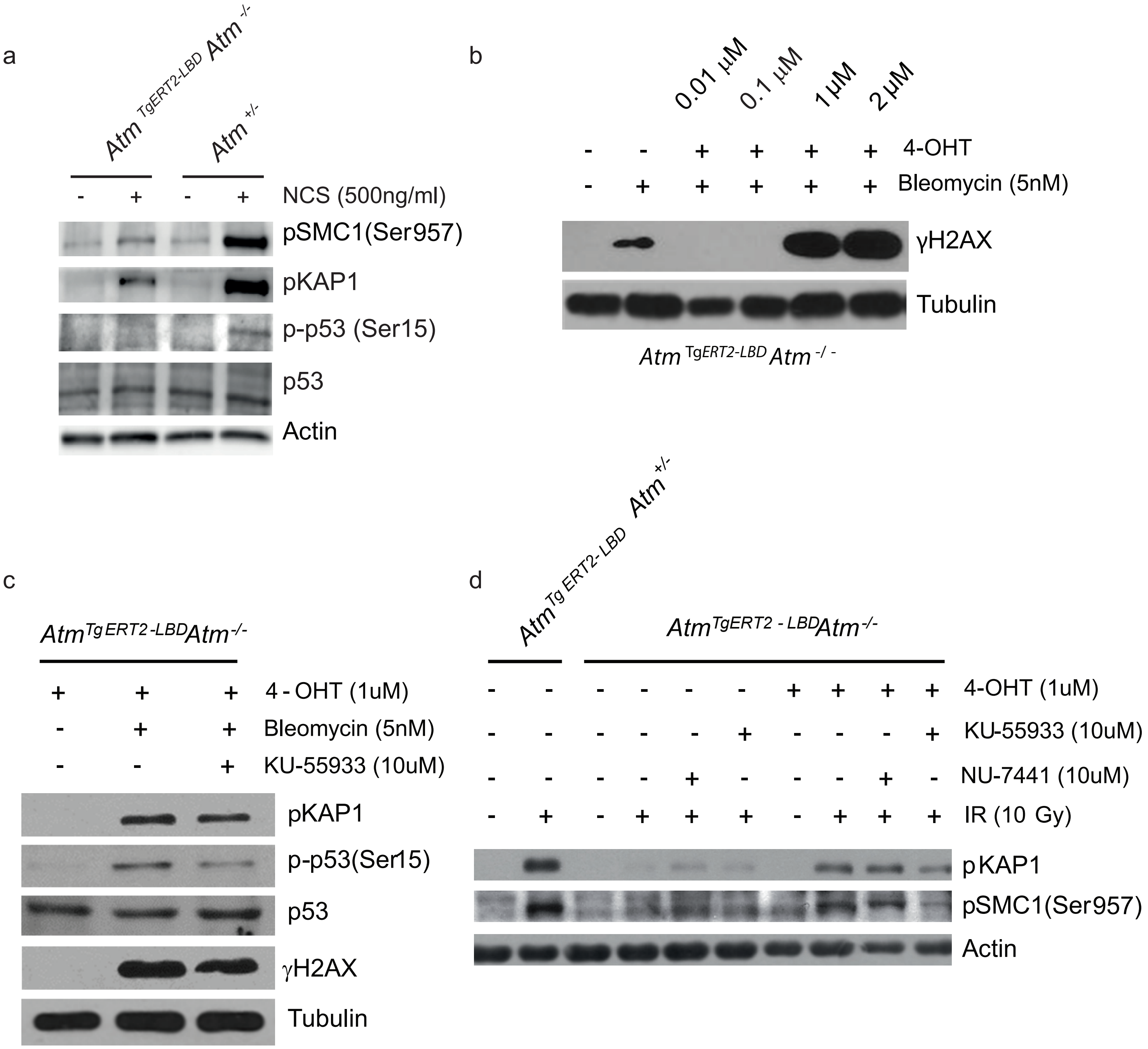

Figure S1. Induced Atm mediates DNA damage response in $Atm^{TgERT2-LBD}Atm^{-/-}$ cells.

a) Western blot analyses of pSMC1, pKAP1 and p-p53 in *$Atm^{Tg\ ERT2-LBD}$ $Atm^{-/-}$* and *$Atm^{+/-}$* thymocytes treated with NCS 500ng/ml for 1h. **b)** Western blot analyses of EFs isolated from *$Atm^{TgERT2-LBD}Atm^{-/-}$* mice and treated with different doses of 4-OHT and 5nM bleomycin. 1µM)4-OHT is the minimum dose found to induce the maximal DNA damage response as evaluated by H2AX phosphorylation. **c)** Atm-dependent DNA damage response was evaluated by phospho-Kap1, -p53 and -H2AX detection in *$Atm^{TgERT2-LBD}Atm^{-/-}$* EFs after 1µM 4-OHT and 5nM bleomycin treatment in the presence or absence of the Atm inhibitor, KU55933. **d)** MEFs from *$Atm^{TgERT2-LBD}Atm^{-/-}$* embryos in which Atm was reactivated with 1µM 4-OHT and from *$Atm^{TgERT2-LBD}Atm^{+/-}$* embryos were treated or not with 10Gy IR. Extracts were prepared 1h after-irradiation and revealed for pSMC1 and pKAP1 antibodies.
MEFs were pre-incubated for 1h with Atm (KU55933) and DNA-PK (NU7026) inhibitors.

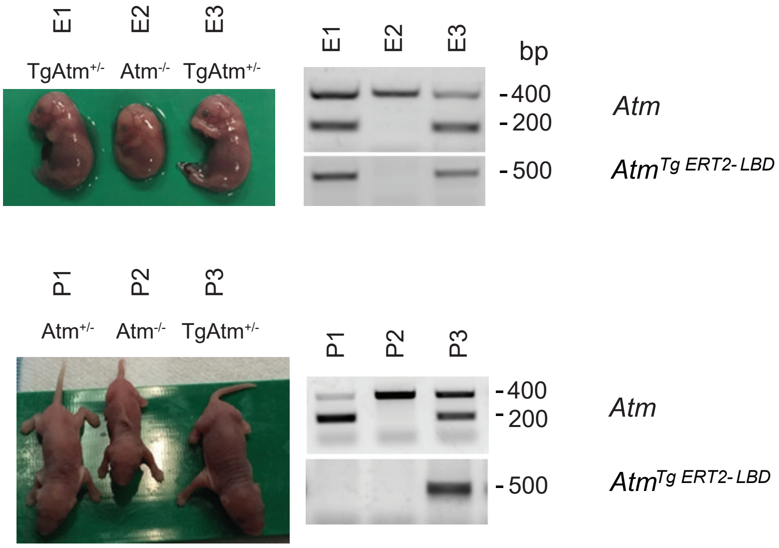

Figure S2. Atm reactivation restores fertility in Atm$^{TgERT2\text{-}LBD}$Atm$^{-/-}$ mice.

Picture of embryos (E17.5 days post coitum; upper panel) and pups (P1-2 days post-partum, lower panel) born from *Atm*$^{TgERT2\text{-}LBD}$*Atm*$^{-/-}$ males injected with tamoxifen crossed with CD1 and Atm$^{+/-}$ females. On the right side representative PCRs for genotyping are shown: Atm wild-type allele (400bp), Atm knockout allele (200bp) and transgene allele (500bp)

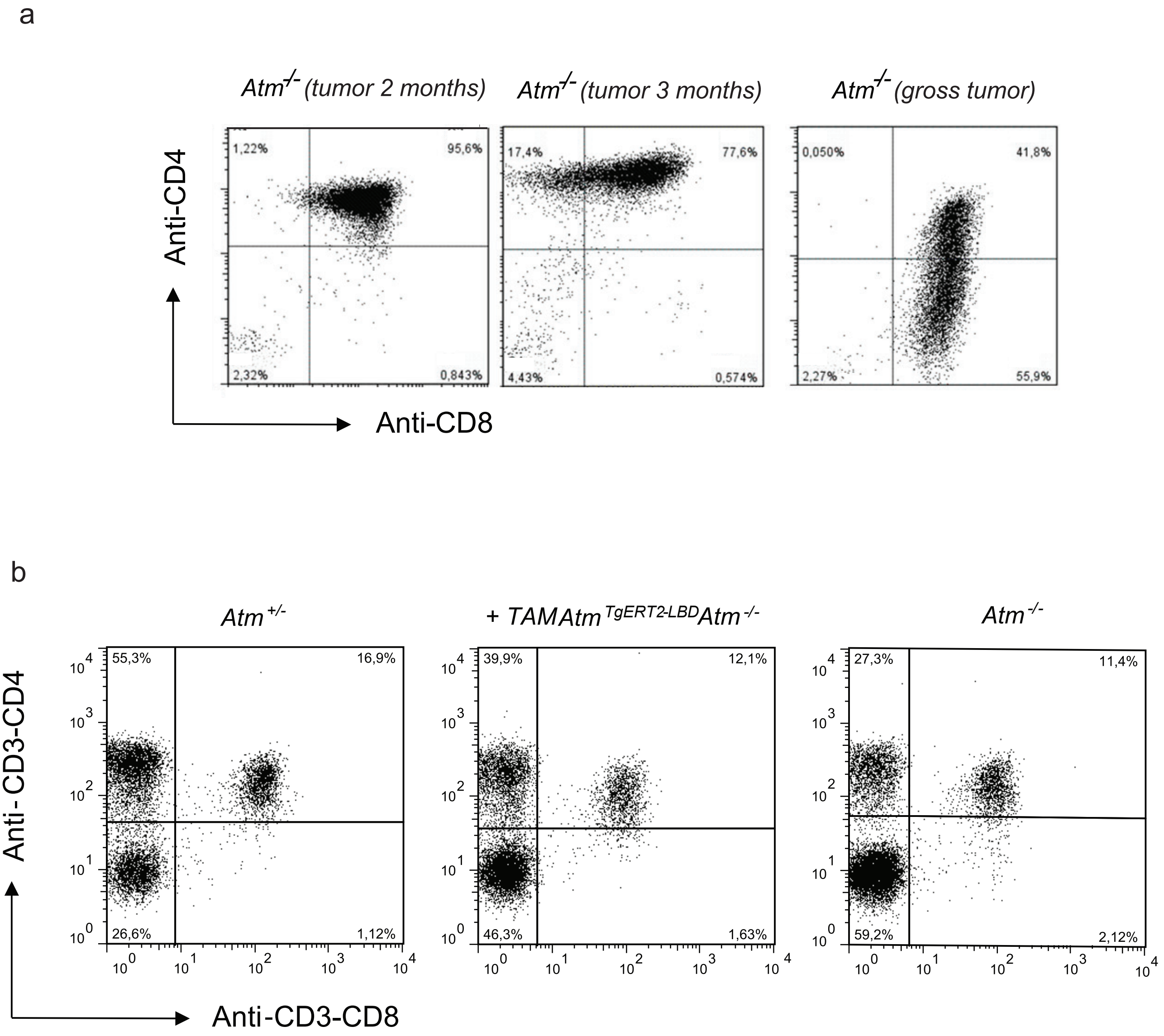

Figure S3. T cell maturation is achieved and maintained in $Atm^{TgERT2-LBD}Atm^{-/-}$ mice after tamoxifen treatment.

a) Flow cytometry analysis of $Atm^{-/-}$ thymocytes collected at different ages and stained for CD4 and CD8 markers. Different stage of thymoma are shown. **b)** Flow cytometry analysis on peripheral blood cells collected from 9 months $Atm^{TgERT2-LBD}Atm^{-/-}$ and $Atm^{+/-}$ old mice after tamoxifen treatment. For $Atm^{-/-}$ mice, blood cells were collected at 2 months of age. Representative CD3-CD4 and CD3-CD8 expression patterns are shown. Data were obtained from independent experiments performed on n=3 mice of each genotype.

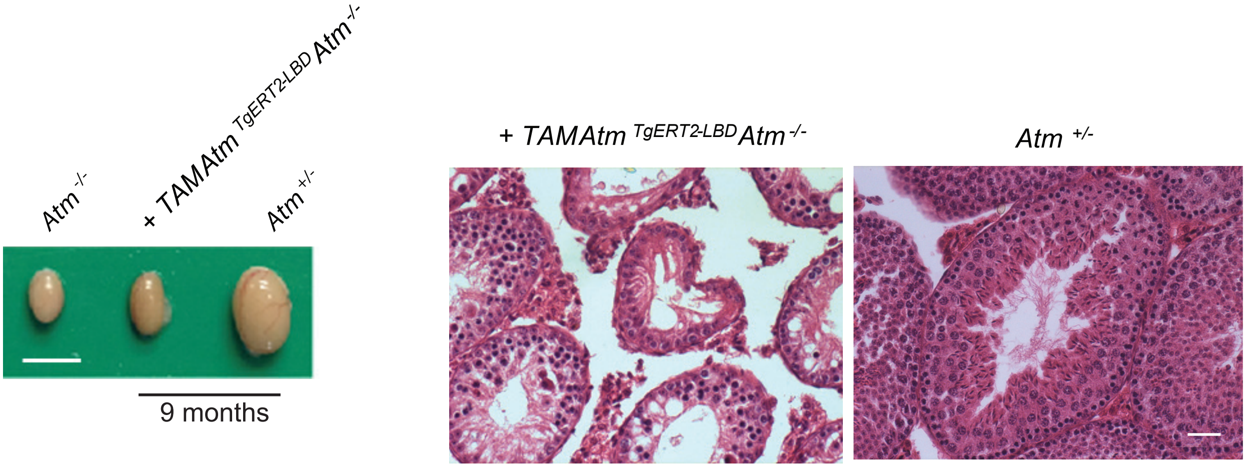

Figure S4. Spermatogenesis is reversible in tamoxifen-treated Atm$^{TgERT2-LBD}$Atm$^{-/-}$ mice .

Testes picture and relative H&E section staining of Atm$^{+/-}$ and Atm$^{TgERT2-LBD}$Atm$^{-/-}$ males 9 months after tamoxifen treatment. Atm$^{-/-}$ testis is shown for comparison. Scale bar=50 mm.
